# MiR-139-5p influences hepatocellular carcinoma cell invasion and proliferation capacities via decreasing SLITRK4 expression

**DOI:** 10.1042/BSR20193295

**Published:** 2020-05-04

**Authors:** Jisiguleng Wu, Tong Zhang, Yubo Chen, Sigaowa Ha

**Affiliations:** 1 Department of Hepatobiliary Surgery, Affiliated Hospital of Inner Mongolia Medical University, Inner Mongolia 010000, China; 2 Department of Geriatric Medicine, Affiliated Hospital of Inner Mongolia Medical University, Inner Mongolia 010000, China

**Keywords:** cell invasion, Hepatocellular carcinoma, microRNAs, proliferation, SLITRK4

## Abstract

The microRNA, miR-139-5p, has been proved to play important roles in regulating tumor progression, including prostate cancer, osteosarcoma, esophageal cancer, and so on, but its correlation of hepatocellular carcinoma (HCC) still remains unclear. Here we found that hsa-miR-139-5p (miR-139-5p) was decreased in HCC samples compared with normal liver tissues, and a lower expression of miR-139-5p was connected to a poorer prognosis. Mechanism study indicated that a decreased/increased miR-139-5p could increase/decrease HCC cells invasion and proliferation capacities via increasing SLITRK4 expression, what’s more, the reverse assays also confirmed the conclusion when we knocked down SLITRK4 in the miR-139-5p low-expression cells. Luciferase assay confirmed that miR-139-5p could directly bind to the 3′UTR of SLITRK4 mRNA to regulate its expression. Together, these findings show the importance of miR-139-5p/SLITRK4 pathway in HCC growth and progression and may provide new targets for us to better arrange the progression of HCC.

## Introduction

Hepatocelluar carcinoma (HCC) is one of the main health problems faced by the public across the word. According to Cancer Statistics, HCC has become the second leading cause of cancer in the world, especially in Africa and Asia [1–3]. What’s more, from the estimated data, the incidence of HCC will rise faster than any other tumors in the U.S.A. in 2019 [1]. HCC is a highly malignant tumor with a high frequency of metastasis and recurrence, which also make it one of the most lethal tumors in the world [4,5]. Every year, HCC will take a large amount of burden in the public health expenditure, therefore it is very essential for us to deeply study the biomarkers related to the progression of HCC and provide novel therapies for better treatments of HCC.

MicroRNAs (miRNAs) belong to the family of non-coding RNAs with 20–24 nucleotides in length. Usually, miRNAs can silence the target genes by post-transcriptional regulation by binding to the 3′ untranslated region (UTR) of target genes [6], and dysregulation of the miRNAs has been proved to have significant correlation with tumor initiation, progression and metastasis [7–10]. MiR-139-5p, located on chromosome 11q13.4, takes part in many important signaling pathway and has been showed to play anti-oncogenic roles in many cancers [11–14]. Therefore, the dysregultion of miR-139-5p in HCC may also lead crucial outcomes in the progression of HCC.

SLIT and NTRK like family member 4 (SLITRK4), located at Xq27.3, belongs to a six-member family of synapse organizers that control excitatory and inhibitory synapse formation by forming trans-synaptic adhesions with LAR receptor protein tyrosine phosphatases (PTPs) [15,16]. SLITRK4 is highly expressed in adrenal, brain and other tissues. Studies have shown SLITRK4 is involved in uterine leiomyosarcoma, brain tumors and neuropsychiatric disorders [17–19].

Here, our study showed that the miR-139-5p expression was decreased in HCC tumor samples, which could increase HCC invasion and proliferation capacity by increasing SLITRK4 expression. Thus, we find a new pathway between miR-139-5p and HCC progression, and may provide novel targets for better therapies for HCC.

## Methods

### Online databases use

The YM500v2 database (http://ngs.ym.edu.tw/ym500v2/index.php), OncomiR database (http://www.oncomir.org/oncomir/search_target_miR.html), Kaplan Meier plotter database (http://kmplot.com/analysis/index.php?p=background) and GEPIA database (http://gepia.cancer-pku.cn/index.html) were used to analyze the clinical correlations and significance of miR-139-5p and SLITRK4 in normal and HCC samples. All the clinical data were gotten from these databases and used according to the instructions and rules of the databases.

### Cell culture

Human HCC SK-HEP-1 and HA22T cells, HEK 293T cells were cultured in Dulbecco’s Modified Eagle’s Medium (DMEM, Invitrogen) with 10% fetal bovine serum (FBS), 1% penicillin/streptomycin and 1% glutamine. All cells were cultured in a 37°C temperate box with 5% (v/v) CO_2_ condition.

### Invasion assay

A total of 1 × 10^5^ HCC cells were seeded into 8-μm transwell chamber (Corning Life Science) with 100 μl diluted Matrigel (BD Corning) coated first in a serum-free medium. The bottom chamber was filled with 700 μl medium (10% FBS). After cultured for 24 h, we collected the transwell chamber for fixation and dyeing of the invaded cells, then observed and counted the cells under the microscope. Each sample was designed in three duplicate chambers and several pictures under different horizons were taken for analysis.

### MTT proliferation assay

A total of 5 × 10^3^ HCC cells were seeded into 24-well plate, and when all the cells adhered to the plate, we marked it as ‘day 0’. We collected the cells of ‘day 0’, ‘day 1’, ‘day 3’, ‘day 5’ for MTT assay. First, we added 500 μl 0.5 mg/ml MTT solution into each hole of the plate and cultured in a 37°C temperate box with 5% (v/v) CO_2_ condition for 2–4 h, then we removed the solution and added 200–500 μl DMSO into the plate. After shaking for 20 min on a shaker, the absorbance of the cells at 570 nm was measured. Each sample was designed in three duplicate chambers and a curve connecting the value of each day was drawn to analyze the proliferation capacity.

### Western blot assay

About 30 μg proteins were loaded into 10% SDS/PAGE gel and then transferred onto a PVDF membrane. After incubated with the primary antibody in a 4°C condition for overnight and secondary antibody at room temperature for 1–2 h, the membrane was visualized with ECL system. The SLTRK4 (ab67315), PCDH7 (ab139274) and GAPDH (6C5) antibodies were purchased from Abcam company. UGT2A3 (PA5-48900) antibody was purchased from Thermo Fisher Scientific company.

### qRT-PCR assay

About 2 μg of RNA was reverse transcribed into cDNA, and the relative expression was calculated by a Bio-Rad CFX96 system. U6 primer was used as internal control to normalize the relative expression. The primers sequences used in the study were as follows: has-miR-139-5p: TCTACAGTGCACGTGTCTCCAGT; U6: Forward: CTCGCTTCGGCAGCACA, Reverse: AACGCTTCACGAATTTGCGT. All the primers were ordered from IDT Company.

### Cell transfection

The pLKO.1-miR-139-5p (oemiR-139-5p), pLKO.1-shSLITRK4 (shSLITRK4), pWPI-SLITRK4 (oeSLITRK4) plasmids, the psPAX2 packaging plasmid and pMD2.G envelope plasmid were transfected into HEK-293T cells using the standard calcium chloride transfection method for 48 h to get the lentivirus soup. The lentivirus soups were collected and concentrated by density gradient centrifugation and used immediately or frozen at −80°C for later use. Lentivirus soups were added into the medium until the cells were 60% confluent. Polybrene (final concentration was 10 μg/ml) was added to improve tansfection efficiency. Twenty-four hours later, the medium was refreshed and transfected with lentivirus soups again. The transfected cells were used for further experiments after checked the tansfection efficiency.

### Luciferase assay

About 1000 bp of SLITRK4 3′UTR was chosen and the potential binding site of miR-139-5p was obtained by using the Targetscan and Genome databases. Based on which, we constructed the wild-type and mutant (the binding site was replaced by GGATCCA) SLITRK4 3′UTR psiCHECK-2 plasmids. Then, the HCC cells were transduced with the wild-type/mutant plasmids and pLKO/oemiR-139-5p or control/miR-139-5p inhibitor (Thermo Fisher Scientific, MH11749). After cultured for 48 h, the cells were collected and the luciferase activity was measured by a dual luciferase reporter assay according to the protocol.

The miR-139-5p inhibitor (MH11749) was purchased from Thermo Fisher Scientific Company. The oemiR-139-5p (pLKO-miR-139-5p) sequence was as follows: Forward: CCGGTCTACAGTGCACGTGTCTCCAGTTTGGATCCGACTGGAGACACGTGCACTGTAGATTTTTG; Reverse: AATTCAAAAATCTACAGTGCACGTGTCTCCAGT CGGATCCAAACTGGAGACACGTGCACTGTAGA.

The wild type/mutant plasmid sequences were:

F1: CAGTAATTCTAGGCGATCGC GTCATGTAATCTTACTTCTT
R1: AGATATTTTATTGCGGCCAGCTACTTACTGATAAATTCACT
F2:CTGCAAAGATGTGTCAAGTA
R2: TACTTACTGATAAATTCACTGGATCCATAATGAGACACCCTTGGAAA

### Statistics

All statistical analysis were conducted by SPSS 22.0 software, and the data were shown as mean ± SD. Differences in mean values between two groups were analyzed by two-tailed Student’s *t* test and the mean values of more than two groups were compared with one-way ANOVA. We thought it as significant difference when *P*≤0.05.

## Results

### The expression and survival rate of miR-139-5p are decreased in HCC samples

**Figure 1:**
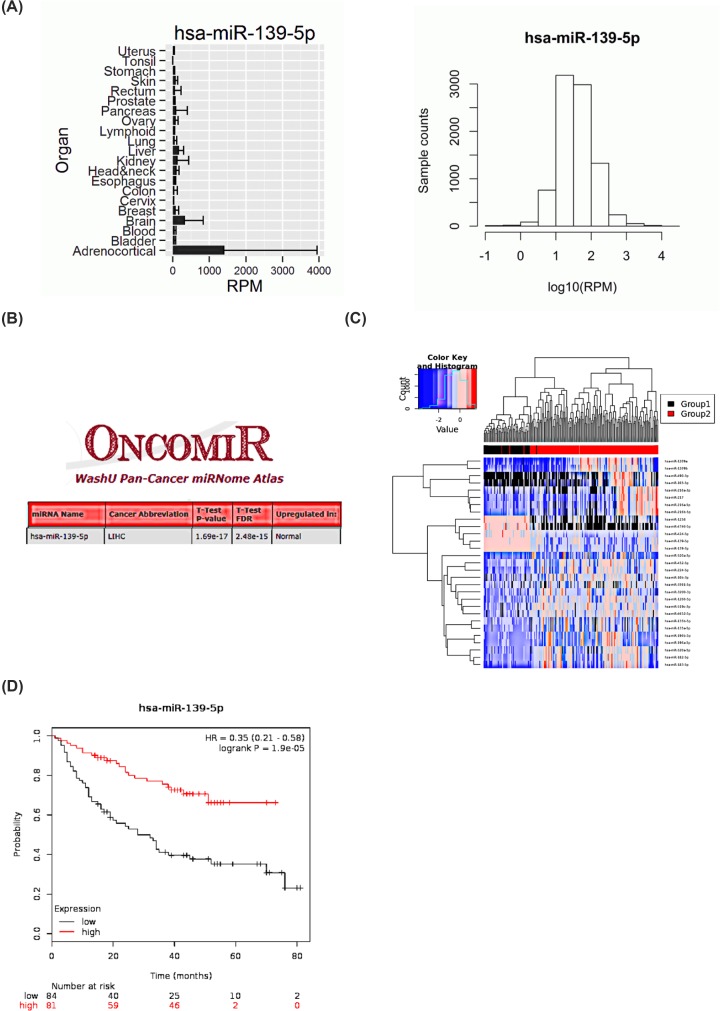
The expression and survival rate of miR-139-5p are decreased in HCC samples (**A**) The YM500v2 database (http://ngs.ym.edu.tw/ym500v2/index.php) was used to checked the miR-139-5p expression in different organs. (**B**) The OncomiR database (http://www.oncomir.org/oncomir/search_target_miR.html) was used to check the miR-139-5p expression in normal liver tissues and tumors. (**C**) Different miRNAs expressions in liver in YM500v2 database. (**D**) The survival rate of different miR-139-5p levels in HCC samples in Kaplan Meier plotter database (http://kmplot.com/analysis/index.php?p=background).

To evaluate the function of miR-139-5p in HCC samples, we first checked the expression of miR-139-5p in liver through the YM500v2 database (http://ngs.ym.edu.tw/ym500v2/index.php). The result showed that the expression of miR-139-5p in liver ranked the third among the overall 21 organs (Figure 1A). We then compared the miR-139-5p expression between normal liver tissues and tumors through the OncomiR database (http://www.oncomir.org/oncomir/search_target_miR.html). The result showed that the expression of miR-139-5p was lower in HCC samples than that in normal tissues (Figure 1B), what’s more, the heat-map in YM500v2 database also confirmed the result (Figure 1C). At the same time, we also assessed the survival rate of different miR-139-5p levels in HCC samples in Kaplan Meier plotter database (http://kmplot.com/analysis/index.php?p=background), and the result proved that a lower expression of miR-139-5p meant a lower survival rate in HCC patients(Figure 1D).

Together, the data from Figure 1A–D show that the expression and survival rate of miR-139-5p decrease in HCC samples.

### MiR-139-5p can decrease the invasion and proliferation capacity of HCC cells

**Figure 2:**
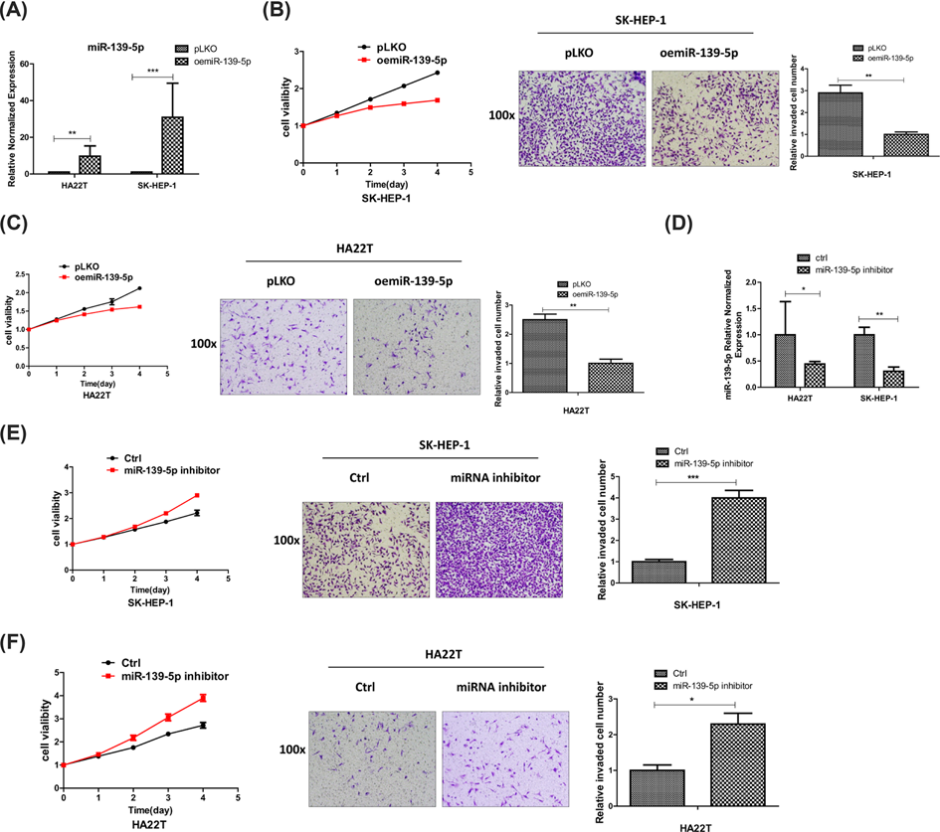
MiR-139-5p can decrease the invasion and proliferation capacity of HCC cells (**A**) Efficiency of overexpression of miR-139-5p in HCC SK-HEP-1 and HA22T cell lines was checked by qRT-PCR assay. (**B** and **C**) The invasion and proliferation capacities were checked by transwell and MTT assays after overexpressing miR-139-5p in HCC cells. (**D**) miR-139-5p expressions were checked by qRT-PCR assay after adding miR-139-5p inhibitor in HCC SK-HEP-1 and HA22T cells. (**E** and **F**) The invasion and proliferation capacities were checked by transwell and MTT assays after adding miR-139-5p inhibitor in HCC cells. *, *P*<0.05, **, *P*<0.01, ***, *P*<0.001.

To evaluate the function of miR-139-5p in HCC cells, we first over-expressed miR-139-5p in HCC SK-HEP-1 and HA22T cell lines (Figure 2A), then we checked the invasion and proliferation capacity of the HCC cells, and the results proved that over-expressing miR-139-5p (oemiR-139-5p) could decrease HCC cells invasion and proliferation capacity (Figure 2B,C). On the other hand, the invasion and proliferation capacity were increased after adding miR-139-5p inhibitor into HCC cells (Figure 2D–F).

Together, the data from Figure 2A–F indicate that miR-139-5p can decrease the invasion and proliferation capacity of HCC cells.

### MiR-139-5p decreases the invasion and proliferation capacities of HCC cells via altering the SLITRK4 expression

**Figure 3:**
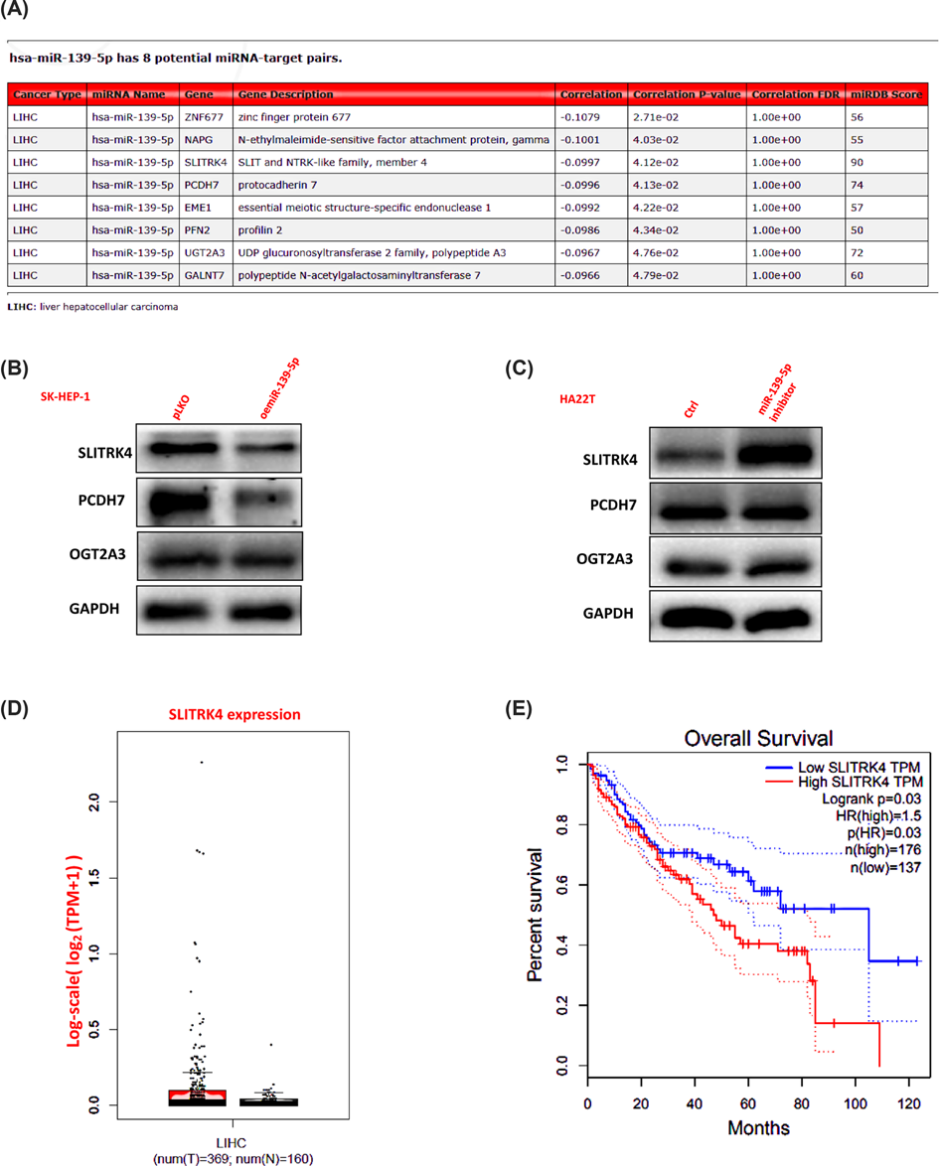
SLITRK4 is the potential target of miR-139-5p in HCC (**A**) The potential downstream genes were predicted by the OncomiR database. (**B** and **C**) Western blots were used to check related protein expressions after oemiR-139-5p or adding miR-139-5p inhibitor into HCC cells. (**D** and **E**) SLITRK4 expression in liver tumors and normal tissues and the survival rate were analyzed by GEPIA database (http://gepia.cancer-pku.cn/index.html).

To study the mechanism of how miR-139-5p decreases the HCC cells invasion and proliferation capacities, we first searched the OncomiR database to predict some potential downstream genes, and from the eight potential targets, we selected three genes for further study (PCDH7, SLITRK4 and UGT2A3, miRDB Score>70) (Figure 3A). We then used Western blots to check the protein expression of the three genes after oemiR-139-5p or adding miR-139-5p inhibitor into HCC cells, and the results that led us to focus on SLITRK4 for its expression were decreased/increased after overexpressing miR-139-5p/adding miR-139-5p inhibitor in HCC cells (Figure 3B,C). We also searched the GEPIA database (http://gepia.cancer-pku.cn/index.html) to check SLITRK4 clinical significance in HCC samples, and the data showed that SLITRK4 expression was increased in HCC samples and a higher SLITRK4 meant a lower survival rate in HCC patients (Figure 3D,E).

**Figure 4:**
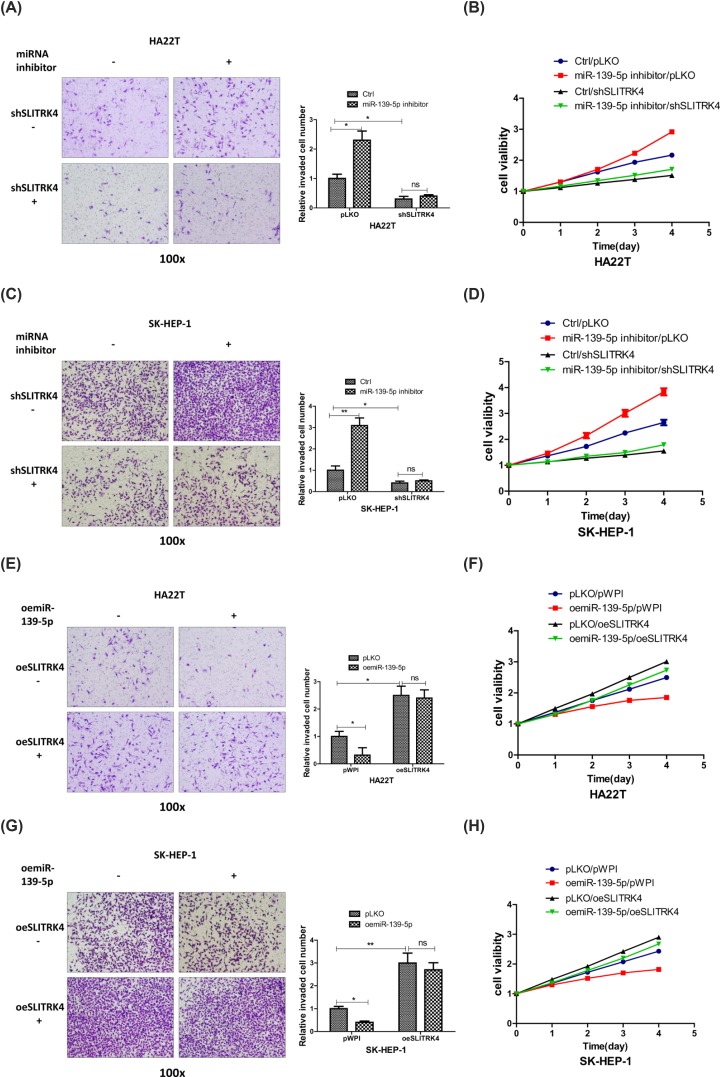
MiR-139-5p decreases the invasion and proliferation capacities of HCC cells via altering the SLITRK4 expression (**A–D**) Invasion and proliferation capacities were measured by transwell and MTT assays after adding miR-139-5p inhibitor/oemiR-139-5p and knocking down/over-expressing SLITRK4 in HA22T cells. (**E**–**H**) Invasion and proliferation capacities were measured by transwell and MTT assays after adding miR-139-5p inhibitor/oemiR-139-5p and knocking down/overexpressing SLITRK4 in SK-HEP-1 cells. *, *P*<0.05, **, *P*<0.01, ***, *P*<0.001.

To assess the function of SLITRK4 under the regulation of miR-139-5p in HCC, we added miR-139-5p inhibitor and knocked down SLITRK4 in HCC cells, and the reverse assays showed that knocking down SLITRK4 could partly reverse miR-139-5p inhibitor’s function to HCC invasion and proliferation, both in HCC SK-HEP-1 and HA22T cells (Figure 4A–D). What’s more, oeSLITRK4 in HCC cells could make the invasion and proliferation capacity more significant (Figure 4E–H), which proved the important function of SLITRK4 in the miR-139-5p pathway.

Together, the results from Figures 3A–E and 4A–H prove that miR-139-5p decreases the invasion and proliferation capacities of HCC cells through SLITRK4 pathway.

### MiR-139-5p directly target 3′UTR of SLITRK4 mRNA to regulate the expression of SLITRK4

**Figure 5:**
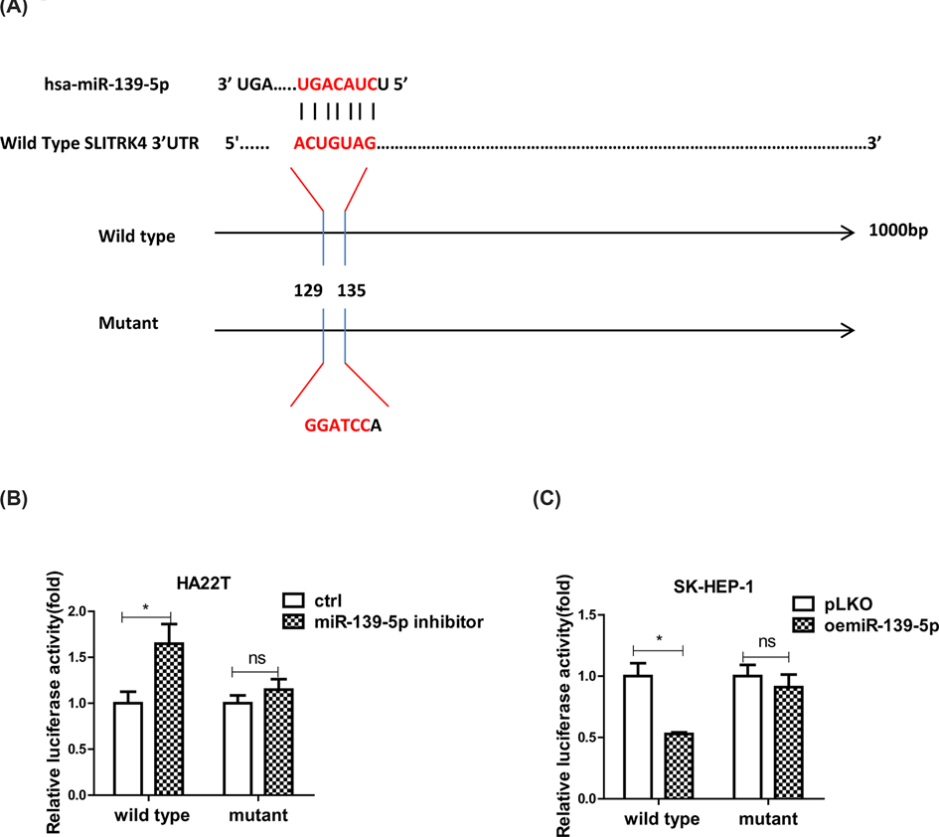
MiR-139-5p directly target 3′UTR of SLITRK4 mRNA to regulate the expression of SLITRK4 (**A**) Pattern diagram of the design of mutant type 3′UTR of SLITRK4. (**B**) Luciferase activitIes were measured when adding miR-139-5p inhibitor in HA22T SLITRK4 wild-type and mutant cells compared with the control cells. (**C**) Luciferase activitIes were measured when overexpressing miR-139-5p in SK-HEP-1 SLITRK4 wild-type and mutant cells compared with the control cells. *, *P*<0.05, **, *P*<0.01, ***, *P*<0.001.

To check how miR-139-5p regulate SLITRK4 expression, we first searched the Targetscan (http://www.targetscan.org/vert_72/) and Genome (http://genome.ucsc.edu/cgi-bin/hgGateway) databases to find out the binding sites of miR-139-5p on the 3′UTR of SLITRK4 mRNA and constructed a 1000 bp wild-type and mutant sequences (Figure 5A), and the results from luciferase assay indicated that the luciferase activity was increased when adding miR-139-5p inhibitor in HCC SLITRK4 wild-type cells compared with the control cells, but the activity in the mutant groups didn’t increase significantly when compared with the control group (Figure 5B). What’s more, the luciferase activity decreased significantly when oemiR-39-5p in HCC cells in wild-type group but not mutant groups (Figure 5C).

Together, the data of Figure 5A–C prove that miR-139-5p directly target 3′UTR of SLITRK4 mRNA to regulate the expression of SLITRK4.

## Discussion

Hepatocellular carcinoma (HCC) is the primary and main type of liver cancer, in 2012, there were 78,250,000 people diagnosed with HCC and 74,550,000 people died of this cancer across the word, and among which, more 50% of the newly diagnose patients and deaths were Chinese [20]. In 2019, there will be approximate 42,030 new cases and 31,780 deaths in United States [1]. HCC is a highly malignant tumor, with high metastatic rate and recurrence rate, and the 5-year survival rate is less than 15% [21]. Chronic hepatitis B virus (HBV) infection is a major risk factor of HCC [22], but today, the promising data showed that approximately 71% of cases were potentially prevented for most of the risk factors were controlled, such as hepatitis B and C viruses, smoking, alcoholism and obesity [23]. Despite this, HCC is still a major health burden worldwide, due to a large population base.

The α-fetoprotein (AFP) and protein induced by vitamin K absence or antagonist-2 (PIVKA-II) are usually used as diagnostic markers for HCC at early stage, but in clinical practice, many HCC patients went late stage when first diagnosed, and HCC patients usually have high post-operative recurrence, many studies have been conducted to find new or more related biomarkers to better predict HCC. He et al. [24] concluded that a history of alcoholism and serum levels of AFP, total protein and c-glutamyl transpeptidase were independently associated with post-operative 1-year recurrence rate in patients with HBV-related HCC who had a single smallprimary tumor (≤3 cm in diameter). Nagai et al. [25] showed that HCC tumor distribution was associated with different molecular species of triglycerides (TGs) and such molecular can be used as biomarkers to character the progression of tumor cells.

Every year, the government has provided millions of money to the studies of HCC, and sorafenib and regorafenib have made great contributions for the therapy of HCC. What’s more, many studies have focused the mechanisms of HCC progression and metastasis [26–29]. Our study based on the clinical samples databases, and identified a new pathway connected to the progression of HCC, is another sample to study HCC progression for better therapy of HCC patients.

microRNAs (miRNAs) are short (20–24 nt) non-coding RNAs that are involved in post-transcriptional regulation of gene expression in multicellular organisms by affecting both the stability and translation of mRNAs. MiR-139-5p, located on chromosome 11q13.4, has been reported to play important roles in many cancers [30–33]. In our study, we found that miR-139-5p expression in liver ranked the third among 21 organs in clinical samples, and usually was decreased in tumors. Mechanism study proved that miR-139-5p can regulated SLITRK4 expression by binding to 3′UTR of SLITRK4 mRNA.

SLITRK4 gene can encode a transmembrane protein, which belongs to the family of SLITRK. The SLITRK4 family members have been reported to include two N-terminal leucine-rich repeat domains that are similar to those in SLIT, the axonal growth-controlling protein, as well as C-terminal regions similar to neurotrophin receptors. SLITRK4 is expressed in many tissues, among which adrenal and brain tissues rank the first two. Until now, only a few studies have reported the function of SLITRK4, many of them are related to nervous system disease [18,19,34,35], and our study is the first time to explore the function of SLITRK4 and its correlation to the progression of HCC. As was shown in Figure 3D,E, SLITRK4 indeed existed in HCC samples, and a higher expression of SLITRK4 was related to a lower survival rate, which proved our study was reasonable. What’s more, our *in vitro* study also proved the importance of SLITRK4 in HCC progression. All of this highlight the importance of SLITRK4 in HCC biological process, and target this SLITRK4 related signaling may provide a new choice for the treatment of HCC.

## Conclusions

In conclusion, our study showed that the miR-139-5p expression was decreased in HCC tumor samples, which could increase HCC invasion and proliferation capacity by increasing SLITRK4 expression. Thus, we find a new pathway between miR-139-5p/SLITRK4 and HCC progression, and target this newly pathway may provide us better therapies for HCC.

## Abbreviations

AFP: α-fetoprotein
HCC: hepatocellular carcinoma
miRNA: microRNA
PTP: protein tyrosine phosphatase
SLITRK4: SLIT and NTRK like family member 4
UTR: untranslated region
